# Supplementary material for: A new interhemispheric teleconnection increases predictability of winter precipitation in southwestern US
Source: Nat Commun. 2018 Jun 13;9:2332. doi: 10.1038/s41467-018-04722-7 (PMC5997984; doi:10.1038/s41467-018-04722-7)
Supplement: Supplementary file 1 — Supplementary Information [file 41467_2018_4722_MOESM1_ESM.pdf]

Supplementary Information

A new interhemispheric teleconnection increases predictability of winter precipitation in southwestern US

by Mamalakis et al.

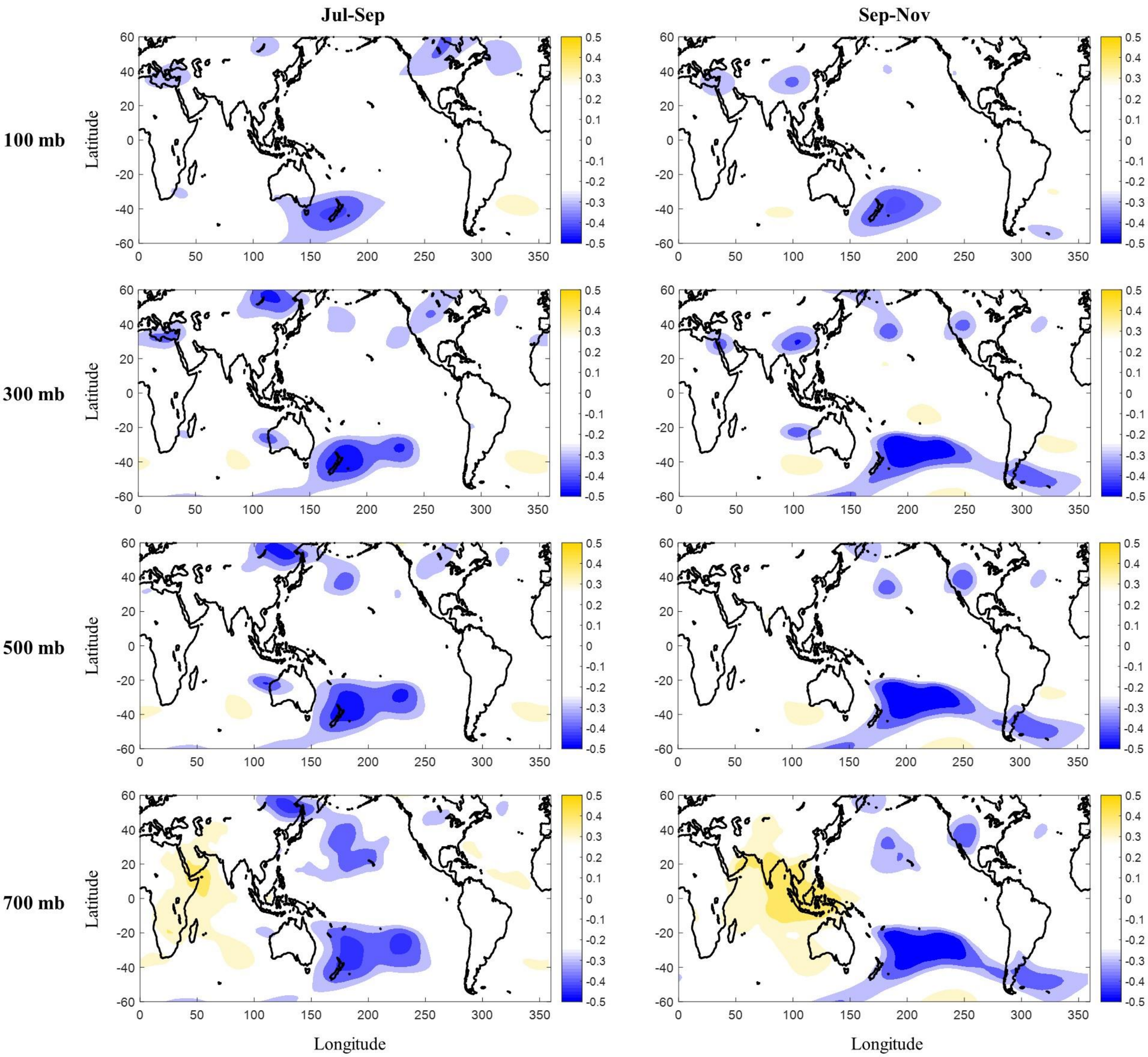

Supplementary Figure 1: NZI had a barotropic structure. Correlation maps between GPH (Jul-Sep and Sep-Nov, at different pressure levels) and the average precipitation in SWUS (Nov-Mar), for 1950-2015 (same results as in Figure 1e-f, but for different pressure levels). White color indicates statistically insignificant correlations (α = 0.05 significance level).

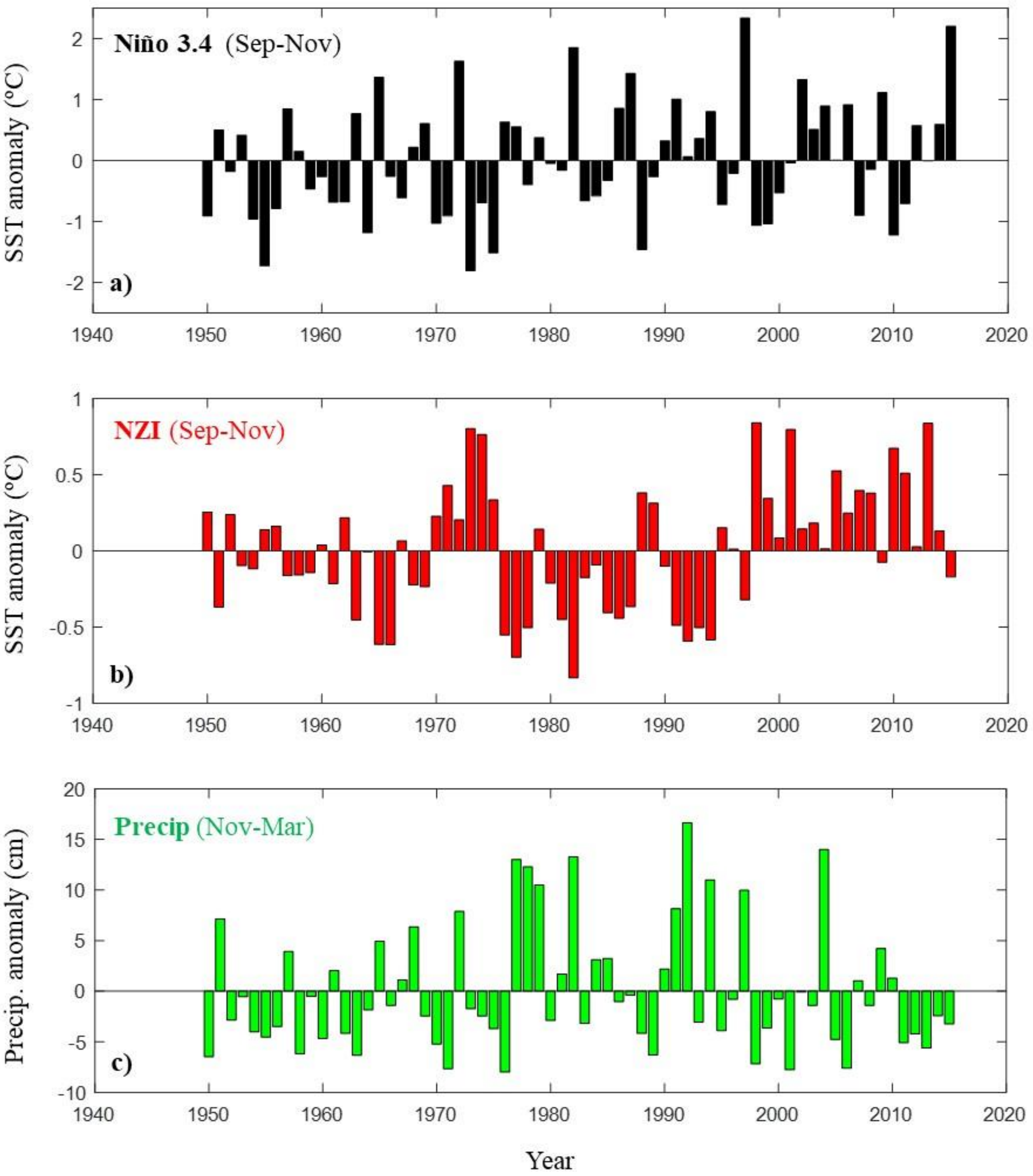

Supplementary Figure 2. Time series of Niño 3.4, and NZI indices (both averaged over Sep-Nov) and precipitation anomaly (Nov-Mar). In (c), each year in horizontal axis refers to the beginning of Nov-Mar.

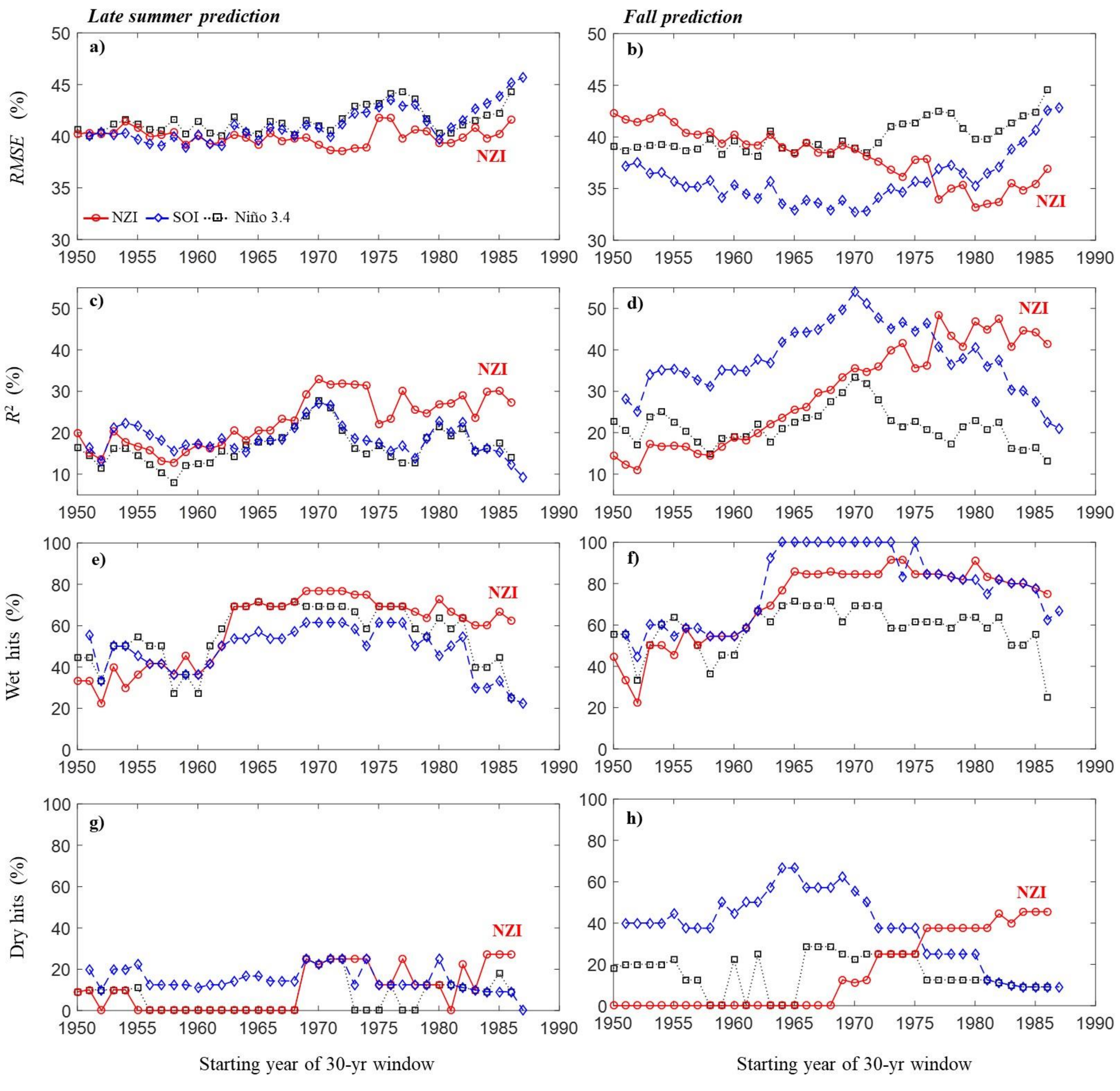

Supplementary Figure 3: **30-year running averages of ENSO and NZI predictive skill for two different lead times indicated the strengthening of NZI after the mid-1970s.** Prediction metrics for 30-yr moving windows are established by comparing models' forecasts and average precipitation amount in SWUS (Nov-Mar): (a)-(b) Standardized *RMSE*; (c)-(d) Coefficient of

determination; (e)-(f) Probability of correctly predicting wet conditions (see section Methods); (g)-(h) Probability of correctly predicting dry conditions.

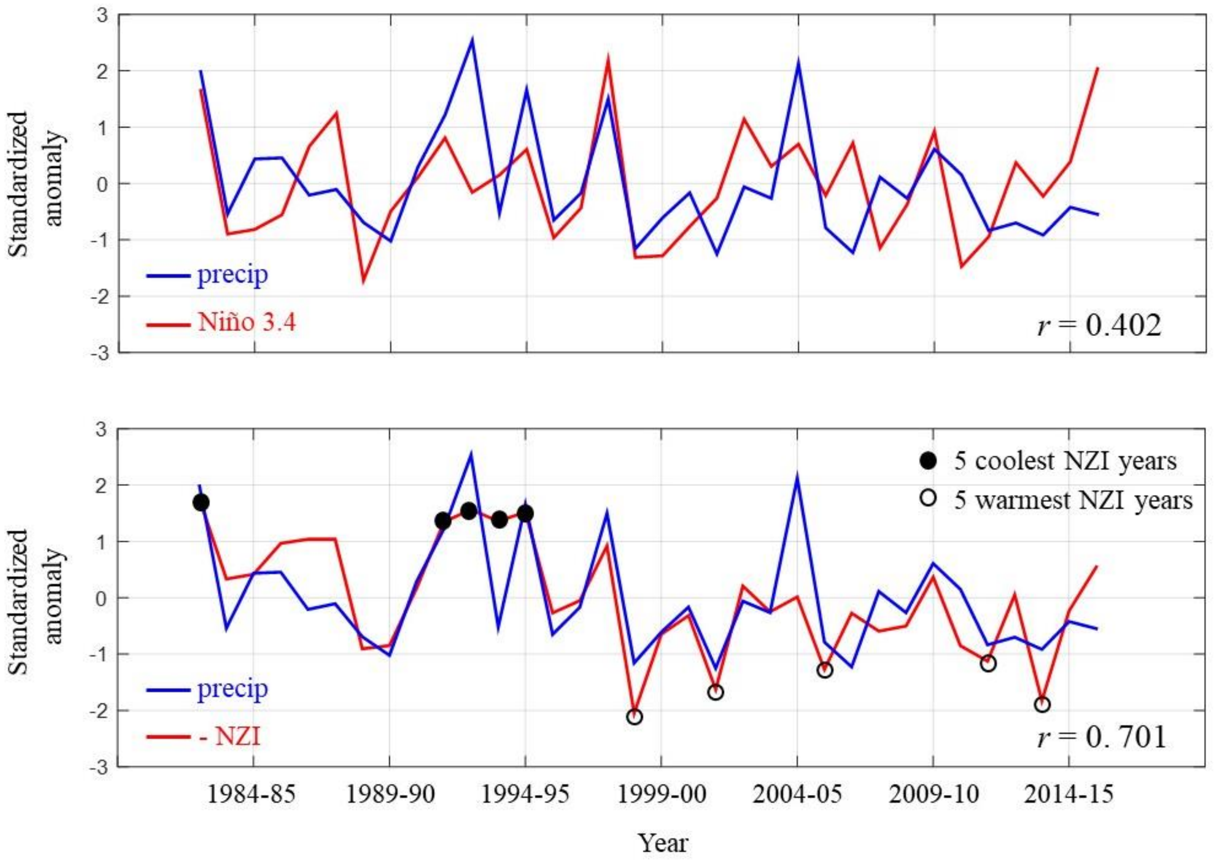

Supplementary Figure 4: **During the last three decades, NZI associated more strongly with the average winter precipitation in the SWUS than Niño 3.4.** NZI and Niño 3.4 anomalies correspond to the Sep-Nov period, while average winter (Nov-Mar) precipitation is computed over climate divisions of significant correlation with Niño 3.4 (see caption of Figure 1). In the lower panel, the 5 coolest/warmest NZI years are indicated (lower/upper ~15%) as well, to be used in Figure 6 and in Supplementary Figure 9.

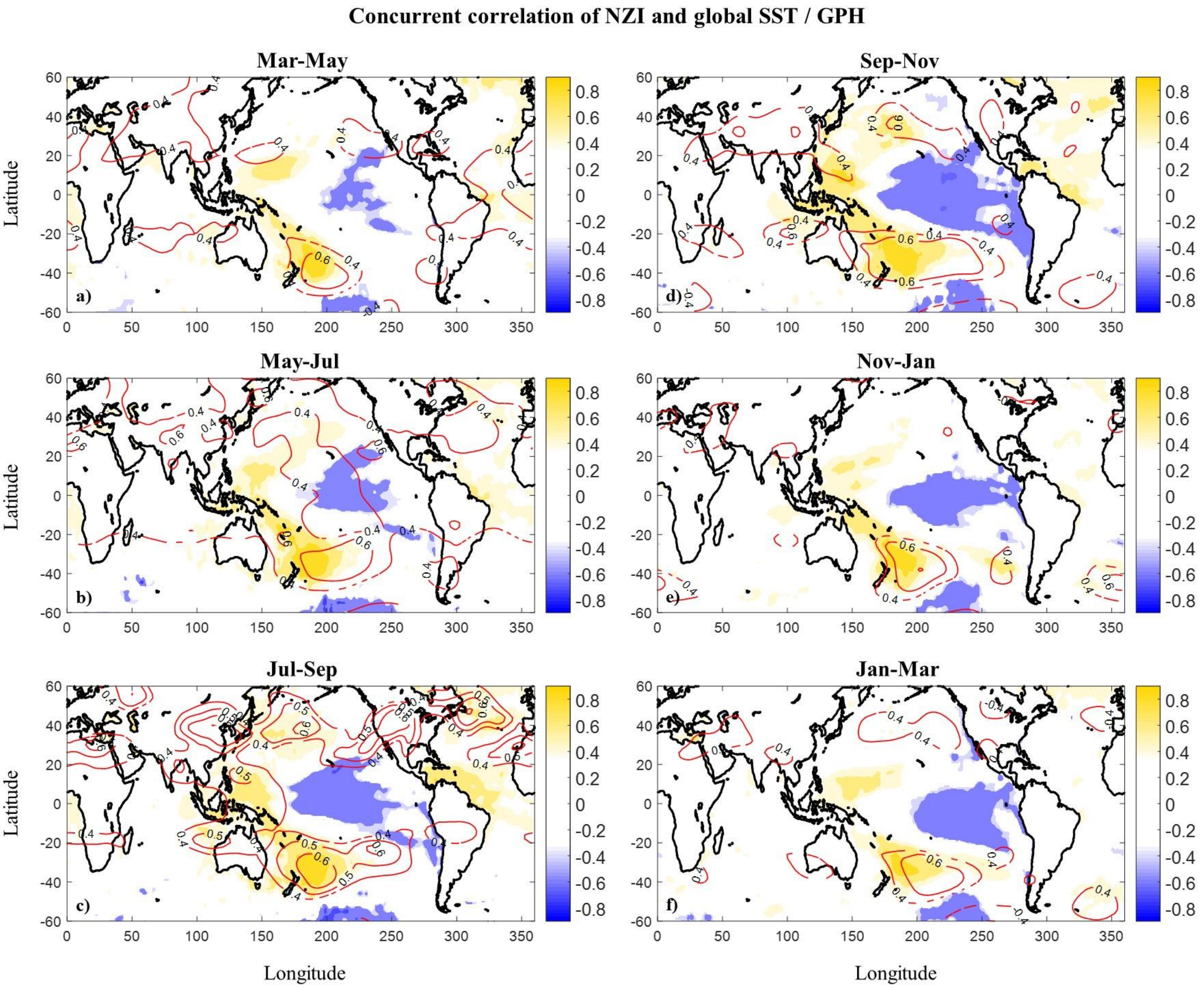

Supplementary Figure 5: SSTs in the region of NZI were closely related with those in the region east of Philippines especially during late boreal summer and fall. Correlation maps between SSTs in the NZI region and global SSTs (shading) and GPHs (400 mb; contours), for 1982-2015. In each panel, NZI, and global SSTs and GPHs are averaged over the same time period, starting Mar-May (top left panel) and ending Jan-Mar (bottom right panel). Only statistically significant ($\alpha = 0.05$ significance level) correlations are shown.

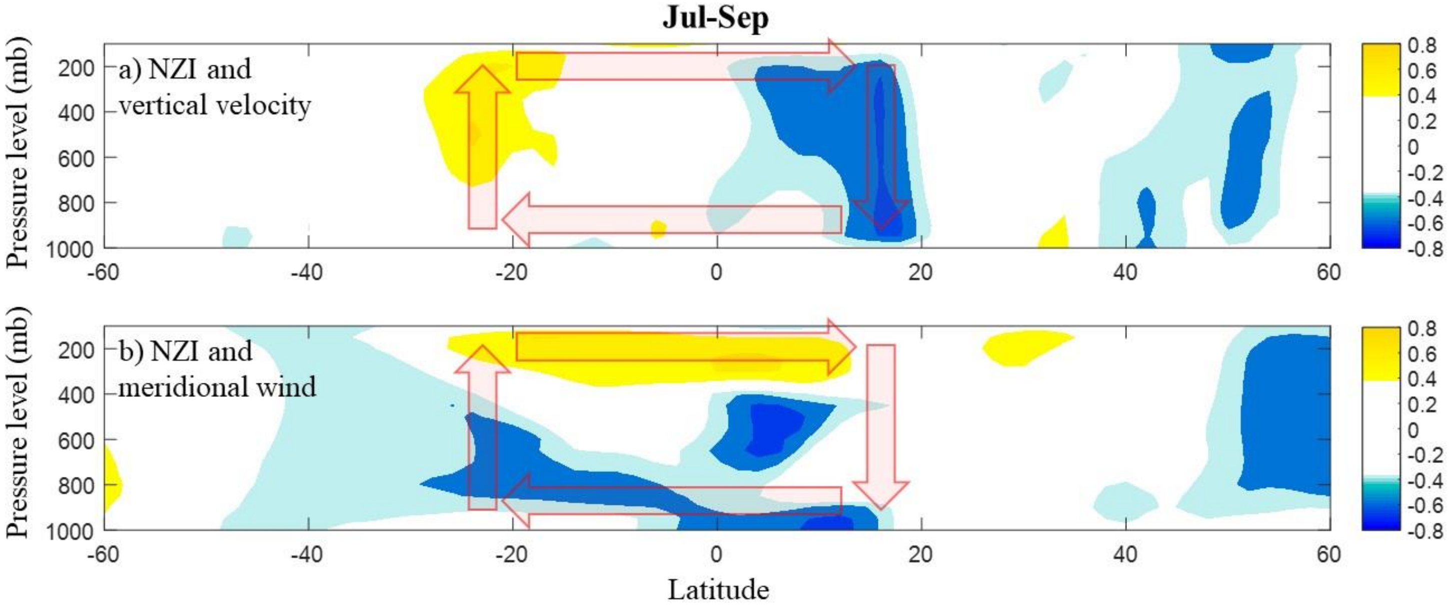

Supplementary Figure 6: Positive SST anomalies in the NZI region weaken the southern Hadley circulation during late boreal summer. (a) Correlation between the NZI (Jul-Sep) and zonal average (70°E-220°E) vertical velocity in Jul-Sep, for 1982-2015 (here, vertical velocity is defined simply as the opposite of omega velocity). Only statistically significant (α = 0.05 significance level) correlations are shown; (b) Same as (a), but for the zonal average meridional wind. In both subplots, the anomalous Hadley circulation is indicated with transparent arrows.

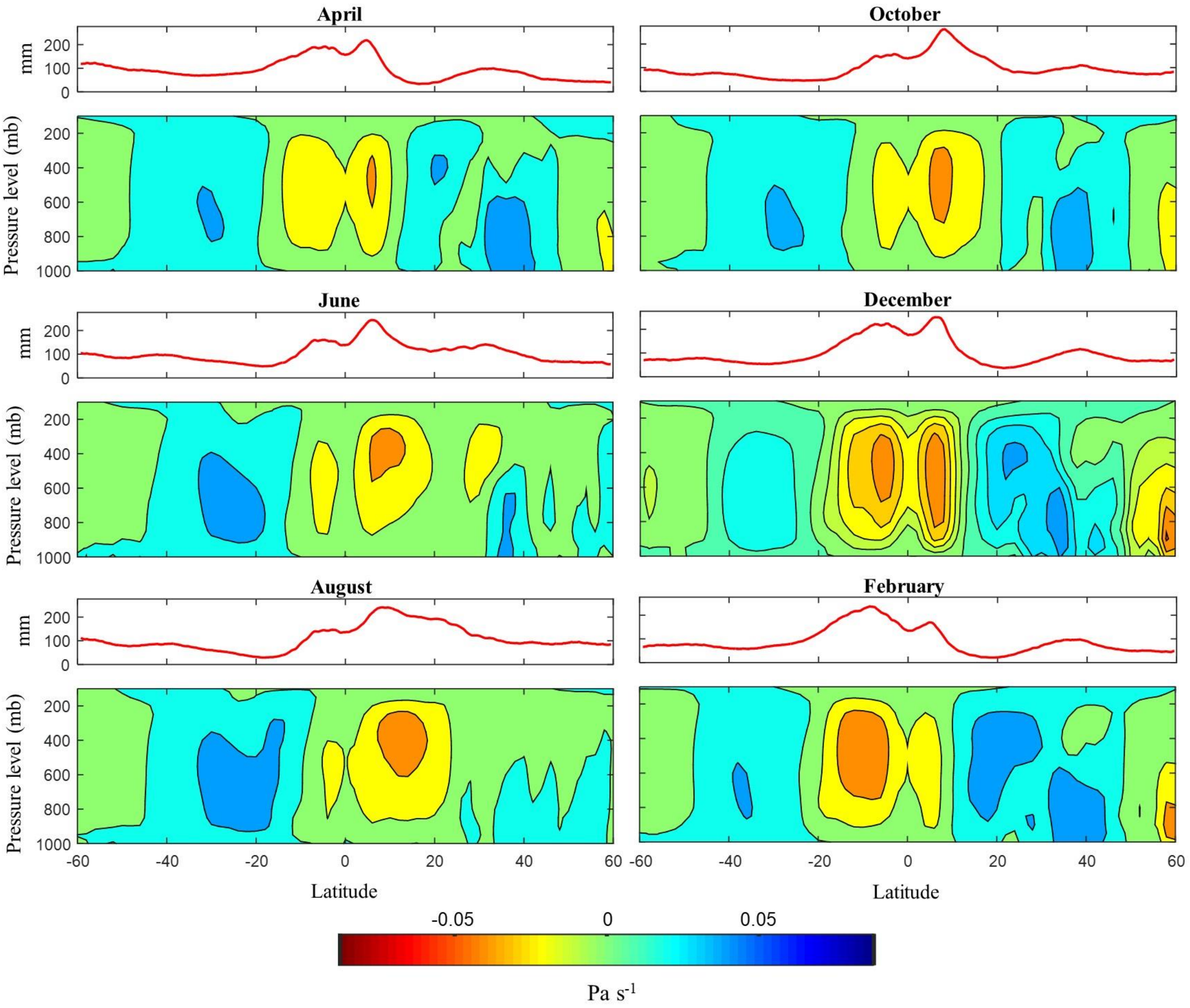

Supplementary Figure 7: **During late boreal summer, the southern Hadley cell expands and connects the area of NZI with the northern hemisphere.** Upper panels: zonal average precipitation (mm) over eastern Asia - western Pacific region (70°E-220°E), for 1982-2015; bottom panels: zonal average omega velocity in Pa s^{-1}, over 70°E-220°E, for 1982-2015. Note that positive (negative) omega velocity corresponds to descending (ascending) motion.

Correlation of -NZI (Jul-Sep) and global SST / GPH

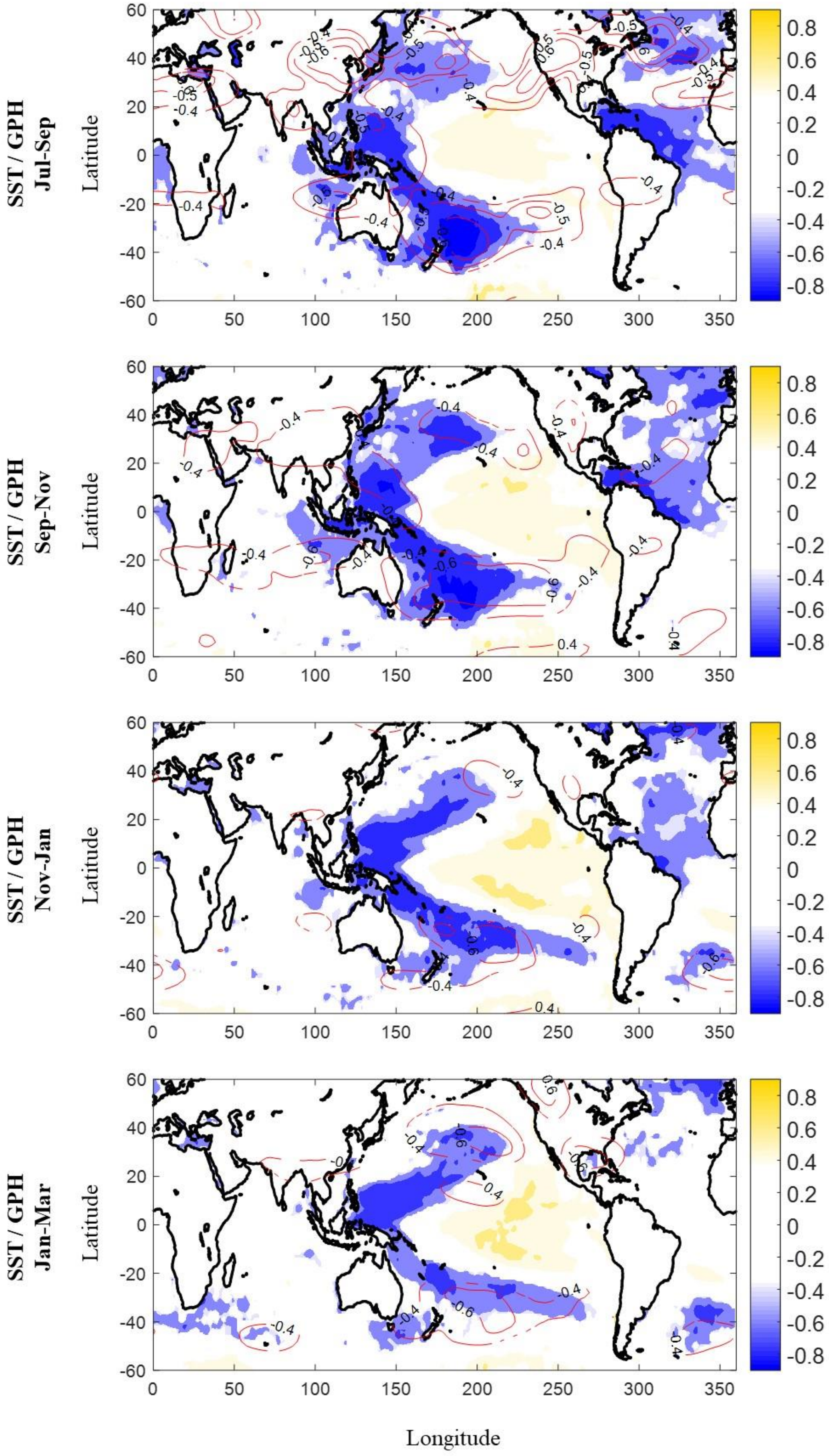

Supplementary Figure 8: NZI anomalies are followed by a northward cascade of SST anomalies in the northern Pacific Ocean starting late summer and ending late winter. Correlation maps between –NZI (averaged over Jul-Sep) and global SST (shading) and GPH (400 mb; contours) in subsequent seasons, derived from reanalysis during 1982-2015. SSTs and GPHs are averaged over 3-month periods, starting Jul-Sep, and ending Jan-Mar. Only statistically significant (α = 0.05 significance level) correlations are shown. We correlate SSTs and GPHs with –NZI, so this figure is directly comparable with results in Figure 4.

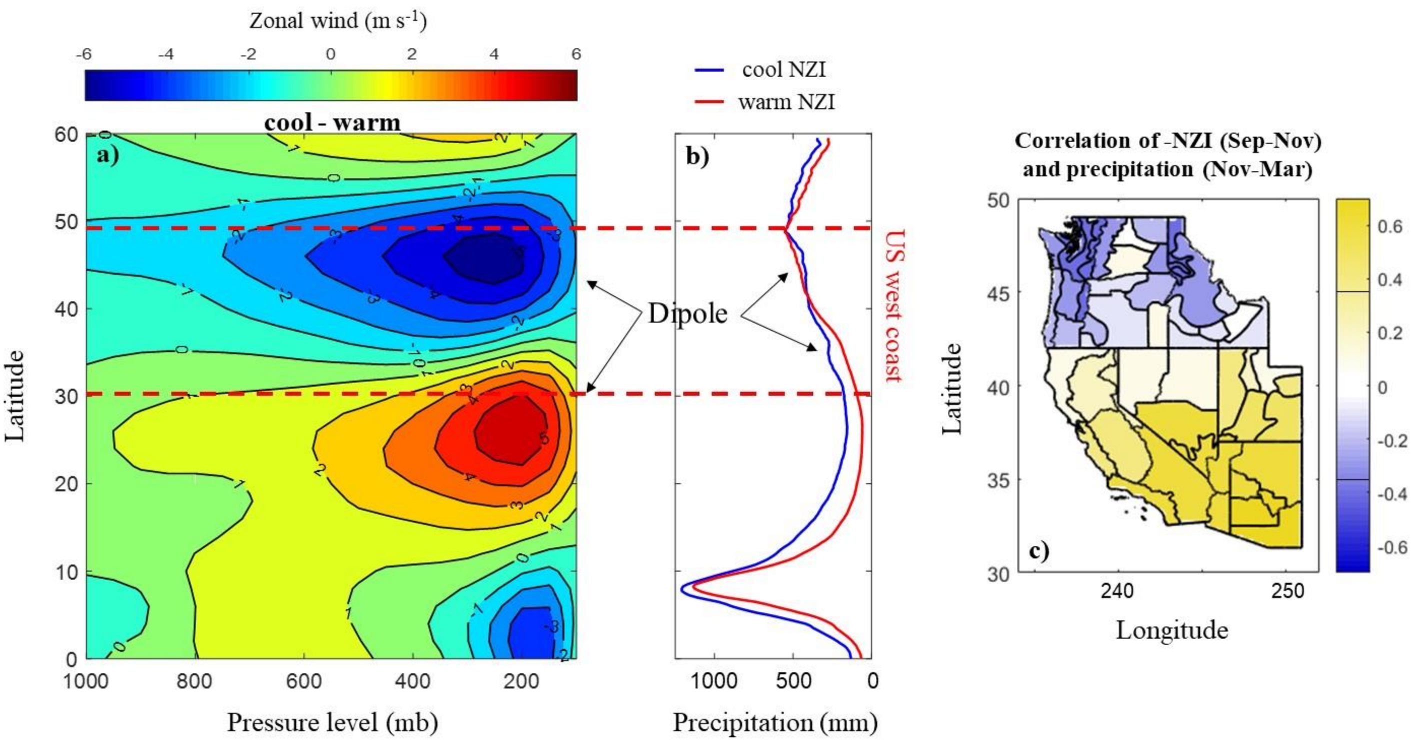

Supplementary Figure 9: NZI was a driver of a dipole of precipitation anomalies along the west coast. (a) Difference of zonal average (220°E-260°E) zonal wind in cool and warm NZI conditions, in m s^{-1}, for Nov-Mar; (b) Zonal average winter precipitation in mm (Nov-Mar), corresponding to the 5 coolest (blue) and 5 warmest (red) NZI years; see Supplementary Figure 4; (c) Correlation of –NZI (Sep-Nov) with winter precipitation (Nov-Mar) in all climate divisions over the west US coast, for 1982-2015. The area between the two solid black lines on the colorbar indicates statistically insignificant (α = 0.05 significance level) correlations.

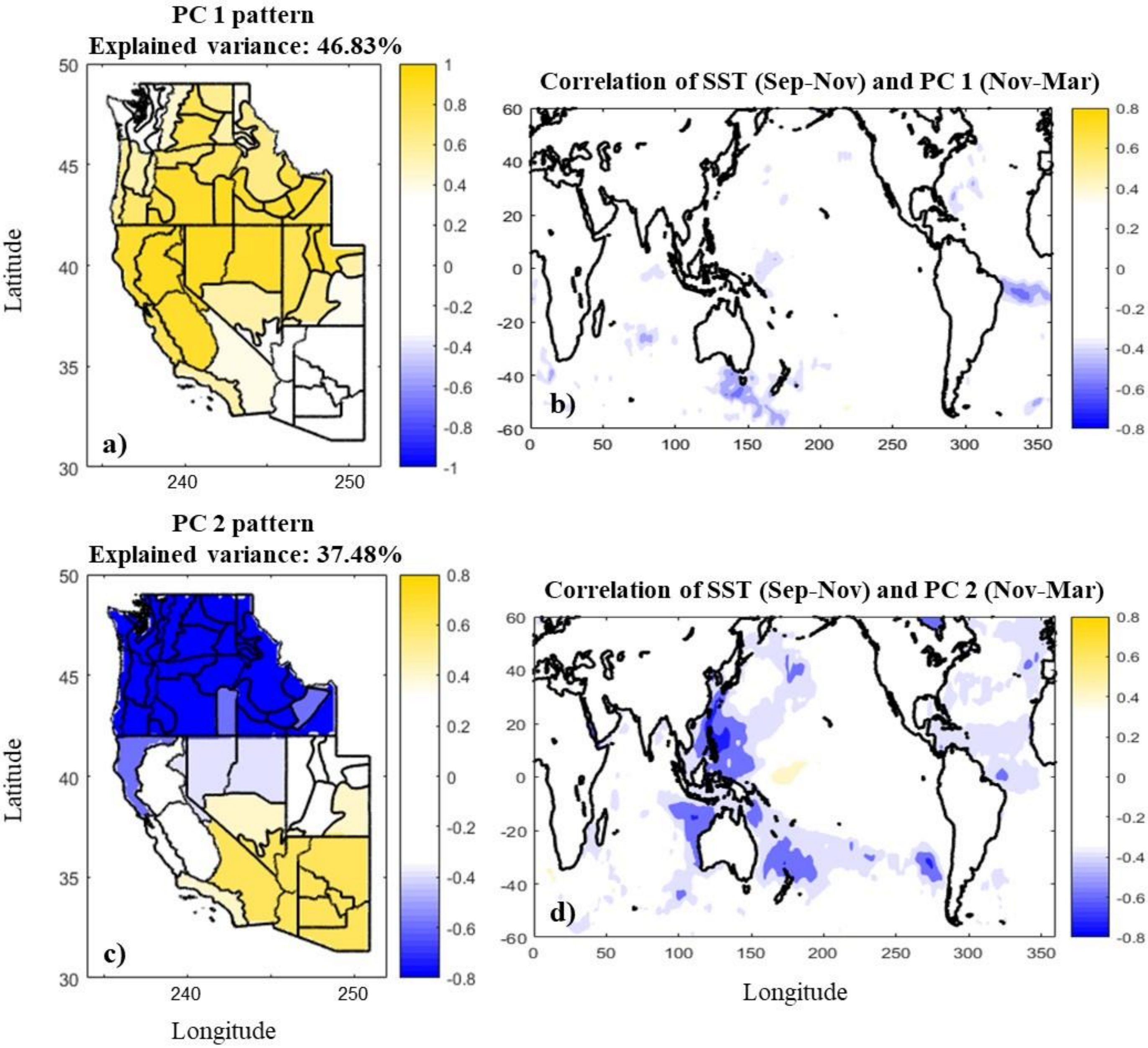

Supplementary Figure 10: **Southwestern Pacific SSTs were strongly correlated with the second principal component of winter precipitation in the western US.** (a) The spatial pattern of the first principal component (PC) of winter (Nov-Mar) precipitation series of all climate divisions in the western US, as determined by its correlation with precipitation in each climate division. Only statistically significant (α = 0.05 significance level) correlations are shown; b) Correlation map between the first principal component and global SST (Sep-Nov), for 1982-2015; (c)-(d) same as (a)-(b), but for the second principal component. Note that PC1 is not related to SSTs, while PC2 exhibits strong statistical dependence with SSTs in the NZI region (correlation on the order of -0.6) but no statistical dependence with SSTs in the ENSO region.

Supplementary Table 1: Effect of non-stationarity in the NZI/ENSO teleconnection strength on the precipitation prediction error. Computed *RMSE*s (cm) of precipitation prediction, when calibration and validation periods overlap (cross validation) and when they do not (***bold italics*** font).

prediction period		1951-1983			1983-2015		
averaging period of predictors		Jul-Sep	Sep-Nov	Nov-Jan	Jul-Sep	Sep-Nov	Nov-Jan
calibration period	1951-1983	5.85	5.39	5.48	***30.75***	***32.45***	***33.97***
	1983-2015	***31.26***	***32.25***	***32.31***	5.97	5.32	5.74
standardized difference (%)		434	498	490	415	510	492
